# Implementing Kidney Disease: Improving Global Outcomes 2024 Through Laboratory-Estimated GFR Across Ages

**DOI:** 10.1016/j.ekir.2025.103767

**Published:** 2026-01-03

**Authors:** Aurélie De Mul, Emilie Bres, Justine Bacchetta, Laurence Chardon, Pierre Letourneau, Sandrine Lemoine, Laurence Derain Dubourg

**Affiliations:** 1 Service de Néphrologie, Dialyse, Hypertension et Exploration Fonctionnelle Rénale, Centre de Référence des Maladies Rénales Rares MAREGE, filière maladies rares ORKID, Hôpital E. Herriot, Hospices Civils de Lyon, Lyon, France; 2 Centre de Référence des Maladies Rénales Rares, Centre de Référence des Maladies Rares du Calcium Et du Phosphore, Filières Maladies Rares ORKiD, OSCAR et ERK-Net, Hôpital Femme Mère Enfant, Bron, France; 3 Faculté de Médecine Lyon Est, Université de Lyon, Lyon, France; 4 INSERM CARMEN 1060 IRIS Team, Lyon, France, Lyon, France; 5 INSERM UMR 1033 LYOS, Lyon, France; 6 Service de Biochimie et Biologie Moléculaire, Laboratoire de Biologie Médicale Multi-Sites, Hospices Civils de Lyon, Bron, France; 7 CNRS UMR 5305, University of Lyon 1, Lyon, France

**Keywords:** BIS1, CKD-EPI, CKiDU25, creatinine, creatinine-based-GFR-estimating-equation, EKFC

## Abstract

**Introduction:**

Chronic kidney disease (CKD) represents a major public health issue, with early detection depending on accurate estimation of glomerular filtration rate (GFR). The 2024 Kidney Disease: Improving Global Outcomes (KDIGO) guidelines recommend age-adapted equations, whereas the 2009 CKD Epidemiology Collaboration (CKD-EPI) equation remains widely used in Europe despite its inapplicability in children and uncertain performance in young adults and the elderly. The objectives of this study were to compare the European Kidney Function Consortium (EKFC) and 2009 CKD-EPI equations for estimated GFR (eGFR)-based CKD staging in a general population (PCrAGE substudy), and to evaluate their performance against measured GFR (mGFR) in a reference cohort (mGFR substudy).

**Methods:**

We analyzed data from 30,104 individuals aged 2 to 99 years with plasma creatinine (PCr) available (PCrAGE). eGFR was calculated using EKFC (all ages), CKD-EPI (adults), and Berlin Initiative Study 1 (BIS1, aged > 70 years). Performance was tested in 4144 mGFR measurements using bias and P30 across age groups and CKD stages.

**Results:**

In PCrAGE, switching from CKD-EPI to EKFC impacted CKD staging (3.2% from stage II to III; 0.4% from stage III tp IV). In the mGFR cohort, all equations showed declining accuracy with advancing CKD stage. EKFC consistently had the lowest bias and highest P30 in adults. CKD-EPI performed poorly in young adults (aged 18–25 years), showing substantial positive bias (∼20 ml/min per 1.73 m^2^) and reduced accuracy. In older adults (aged > 70 years), performance was similar across equations.

**Conclusion:**

EKFC provides a continuous and robust equation for eGFR estimation across the lifespan, offering an advantage over CKD-EPI.

## Graphical abstract

**Figure:**
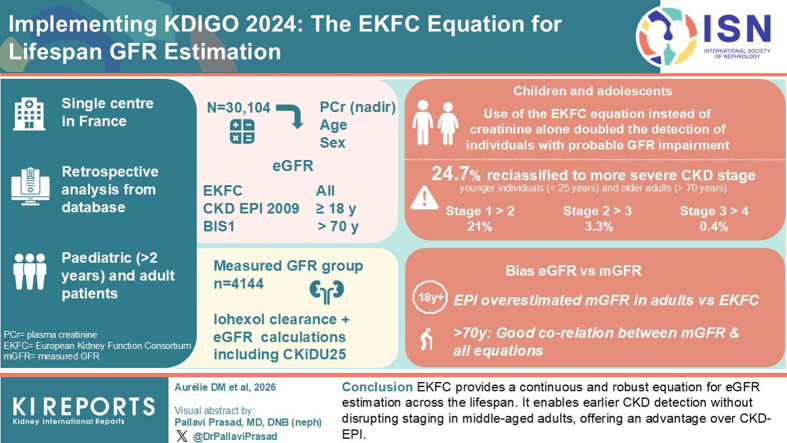

The early diagnosis of CKD is a critical public health priority, because of its rising prevalence and strong associations with cardiovascular risk, morbidity, and mortality.^1^^,^^2^ GFR remains the most reliable marker of kidney function, and together with albuminuria, underpins the diagnosis, staging, and management of CKD. According to KDIGO, CKD is defined as GFR < 60 ml/min per 1.73 m^2^ and severe CKD as GFR < 30 ml/min per 1.73 m^2^.^3^ PCr, the most widely used biomarker for GFR estimation, is influenced by nonrenal factors such as muscle mass, age, sex, and diet, limiting its reliability when interpreted in isolation. To enhance accuracy, several creatinine-based eGFR equations have been developed, including the Cockcroft–Gault equation,^4^ the Modification of Diet in Renal Disease equation,^5^ and the 2009 CKD-EPI equation.^6^ Since 2013, KDIGO guidelines have recommended eGFR reporting with every creatinine measurement.^7^ The 2009 CKD-EPI equation was globally adopted until its 2021 revision, which removed the race coefficient.^8^ However, the revised version has been shown to underperform in European populations, prompting many European laboratories to retain the 2009 version.^9^, ^10^, ^11^ Although CKD-EPI 2009 performs adequately in middle-aged adults, its accuracy decreases in individuals aged < 25 years or > 70 years, potentially leading to misclassification of CKD severity.^12^^,^^13^ Moreover, it is not applicable in children, for whom specific equations have been developed.^14^^,^^15^ Reflecting these concerns, the 2024 KDIGO guidelines now advocate the use of population-tailored GFR equations.^3^ In children and young adults, the CKD in Children equation^14^ and its extension for individuals aged < 25 years (CKD in Children under 25 [CKiDU25])^15^ have been developed; however, these require height measurements and involve a change in formula at the age of 25 years. For elderly patients, the BIS1 equation offers an alternative, though its performance compared with CKD-EPI remains variable.^16^, ^17^, ^18^, ^19^, ^20^, ^21^ More recently, the EKFC equation was introduced,^22^ derived from a large multiethnic European cohort to provide continuous, age-adapted GFR estimation from childhood to old age, avoiding abrupt transitions at critical age thresholds.^22^, ^23^, ^24^ This equation was also developed with the inclusion of height (EKFC-height). The choice of GFR estimation equation has direct implications for patient care and public health, influencing CKD staging, treatment decisions, and health care resource allocation. This study aimed to do the following: (i) compare the clinical impact of implementing the EKFC versus the 2009 CKD-EPI equation for eGFR estimation and CKD staging in a general population undergoing routine PCr testing over 3 months (PCrAGE substudy), and (ii) assess the performance of commonly used GFR equations (EKFC [EKFC, age dependent and EKFC-height], CKiDU25, BIS1, and CKD-EPI) in a local cohort of selected patients with available mGFR values (mGFR substudy).

## Methods

All procedures were conducted in accordance with the ethical standards of the institution, the 2013 Declaration of Helsinki and its subsequent amendments, or equivalent ethical guidelines for single-center retrospective studies. Ethical approval for this retrospective study was granted by the local institutional review board (Comité d'Éthique des Hospices Civils de Lyon, approval number 24-5201, session October 2024).

### Patients With PCr Determination and eGFR Using Various Formulas (PCrAGE Substudy)

We retrieved data from the laboratory database of the centralized hospital laboratory (Biochemistry and Molecular Biology Department, Hospices Civils de Lyon, France), including data on sex, age, and PCr measurement for all patients aged > 2 years who underwent PCr testing between November 1, 2023, and January 31, 2024. Because the database contains multiple serial measurements for some patients, the data were filtered to retain only the lowest PCr value per patient over the study period. The lowest creatinine value was selected to avoid over diagnosing CKD in hospitalized patients with acute kidney injury. After filtering, the dataset was reduced from 78,778 to 30,104 individuals. The patients were subsequently stratified into subgroups based on sex and age. PCr was measured using an enzymatic method traceable to isotope dilution mass spectrometry (Architect c, Abbott Diagnostics) traceable to the National Institute of Standards and Technology (NIST SRM 967 and NIST SRM 914) with a declared analytical coefficient of variation of ± 4.21%. Reference values for PCr by sex and age were provided by the manufacturer.^25^

The eGFR was calculated for all patients using the EKFC equation. For patients aged > 18 years, eGFR was calculated using the 2009 CKD-EPI equation. In addition, for patients aged > 70 years, eGFR was estimated by using the BIS1 equation. The CKiDU25 and the EKFC-height equations could not be calculated because of the unavailability of height measurements in routine laboratory data. In Supplementary Table S1, we summarize the different GFR estimation formulas used.

### eGFR Using Different Formulas in Patients With an mGFR (mGFR Substudy)

We analyzed data from 4144 consecutive patients who underwent a GFR measurement (mGFR) using iohexol clearance as part of routine renal evaluations for suspected underlying kidney pathology between January 2014 and October 2023.

#### Anthropometric and Biochemical Data Collection

Demographic and clinical data, including age, sex, body weight, height, body surface area, body mass index, PCr, and mGFR, were extracted from patients’ medical records.

Body surface area was calculated using the DuBois formula.^26^ PCr was measured in an overnight fasting patients on the same day as the iohexol determination using an enzymatic method traceable to isotope dilution mass spectrometry (Architect c, Abbott Diagnostics). Reference values for PCr creatinine by sex and age were provided by the manufacturer.^25^

#### GFR Estimation and Measurement

eGFR was calculated using different formulas, as outlined in Supplementary Table S1. The CKiDU25 equation and EKFC-height for patients aged 2 to 25 years, the 2009 CKD-EPI equation for patients aged ≥ 18 years, the BIS1 equation for patients aged > 70 years, and the EKFC equation (age-dependent) for all patients.

Iohexol plasma clearance assessment was performed according to a standardized technique, with a single-bolus injection. First, a bolus of 5 ml in adults and about 5 ml/1.73 m^2^ iohexol (Omnipaque 300; GE Healthcare SAS, Vélizy-Villacoublay, France) was injected, and the actual dose injected was determined by weighing the syringe before and after injection. Second, blood was sampled at 120, 180, and 240 minutes after injection when the eGFR was > 40 ml/min per 1.73 m^2^, with an additional 360-minute sample for lower eGFRs. Plasma iohexol concentrations were determined using high-performance liquid chromatography following the high analytical performance method published by Cavalier *et al.*^27^ External quality control was provided by Equalis (Uppsala, Sweden) every 3 months. mGFR was calculated from the slope of plasma concentrations using a 1-compartment model corrected with the Bröchner-Mortensen formula, and the results were expressed per 1.73 m^2^ of body surface area.^26^

### Statistical Analysis

#### PCrAGE Substudy

Results were expressed as median and interquartile range. Agreement between EKFC, BIS1, and CKD-EPI (considered the reference) according to age groups were expressed as median bias with 95% confidence interval (CI), and as the number of patients reclassified as < 60 or < 30 ml/min per 1.73 m^2^. Pairwise McNemar tests were used for comparisons. The full distribution of eGFR was displayed using violin plots according to age.

The agreement on frequency of the KDIGO categories according to each eGFR equation was assessed using Cohen’s kappa (κ) with 95% CI. Kappa coefficient was interpreted as follows: ≤ 0.20, slight agreement; 0.21 to 0.40, fair agreement; 0.41 to 0.60, moderate agreement; 0.61 to 0.80, substantial agreement; 0.81 to 1, almost perfect agreement.

#### mGFR Substudy

Results were expressed as median and interquartile range. The agreement between mGFR and eGFR was assessed by calculating the median absolute bias (eGFR − mGFR, ml/min per 1.73 m^2^; 95% CI), the imprecision (SD [P25−P75]) and accuracy with P30 (95% CI) and P10 (95% CI) (i.e., the proportion of eGFR values within ±30% and ±10% of mGFR, respectively). Pairwise McNemar tests were used for comparisons. Multiple comparisons were assessed using the Kruskal-Wallis test followed by *ad hoc* posttests. The full distribution of eGFR and mGFR was displayed using violin plots according to age. We stratified according to the stage of CKD based on the average of the mGFR and the eGFR, using age-appropriate reference equations (CKiDU25 for patients aged < 25 years and EKFC for individuals aged ≥ 25 years) as previously described.^28^

The eGFRs derived from different equations were compared using Bland-Atman plots. All analyses were performed using GraphPad Prism (version 8; GraphPad Software, Inc, San Diego, CA). A *P*-value < 0.05 was considered statistically significant.

## Results

### PCrAGE Substudy

In Table 1, we present the characteristics of the study population, alongside eGFR results calculated using EKFC (all patients), CKD-EPI (adults aged > 18 years), and BIS1 (adults aged > 70 years). In addition, it provides a detailed comparison of median biases among the 3 equations across various adult age groups and outlines the proportion of patients reclassified into different CKD stages.

**Table 1:** Description of the whole population and each age group characteristics of patients with PCr measurement (PCrAGE substudy)

Age Group	All	≥ 2 to < 12 yrs	≥ 12 to < 15 yrs	≥ 15 to < 18 yrs	≥ 18 to < 25 yrs	≥ 25 to < 40 yrs	≥ 40 to < 55 yrs	≥ 55 to < 70 yrs	≥ 70 yrs
*n*	30104	2672 (8.9)	937 (3.1)	1131 (3.8)	2359 (7.8)	4891 (16.2)	4904 (16.3)	5667 (18.8)	7543 (25.1)
Males (%)	14993 (49.8)	1496 (56.0)	458 (48.9)	544 (48.1)	974 (41.3)	2157 (44.1)	2483 (50.6)	3131 (55.2)	3750 (49.7)
Age (yrs)	49.9 (43.8)	6.7 (5.3)	13.7 (1.5)	16.5 (1.5)	21.6 (3.4)	32.3 (7.4)	47.9 (7.8)	62.5 (7.7)	79.7 (11.2)
PCr (μmol/l)	66.0 (30.0)	33.0 (14)	49.0 (15.0)	59.0 (18.0)	63.0 (21.0)	64.0 (23.0)	67.0 (25.0)	71.0 (31.0)	77.0 (39)
CKD-EPI (ml/min per 1.73 m^2^)					126.5 (16.6)	116.8 (18.3)	102.6 (21.7)	90.6 (25.8)	72.2 (31.6)
EKFC (ml/min per 1.73 m^2^)	92.0 (38.3)	110.6 (14.7)	112.1 (13.1)	109.1 (17.1)	109.9 (13.9)	110.3 (15.8)	99.7 (20.4)	84.1 (23.1)	63.9 (27.1)
BIS1 (ml/min per 1.73 m^2^)									58.9 (24.6)
PCr > ULN (%)	4060 (13.5)	256 (9.6)	69 (7.4)	29 (2.6)	53 (2.4)	254 (5.2)	502 (10.2)	941 (16.6)	1956 (25.9)
EKFC-CKD-EPI (ml/min per 1.73 m^2^)					−16.3 [−16.5 to −16.0]	−6.1 [−6.2 to −5.8]	−2.1 [−2.2 to −2.1]	−5.6 [−5.6 to −5.5]	−7.7 [−7.8 to −7.5]
BIS1-CKD EPI (ml/min per 1.73 m^2^)									−9.3 [−9.5 to −9.1]
EKFC-BIS1 (ml/min per 1.73 m^2^)									−1.9 [−2.1 to −1.8]
*N* (%) reclassified with EKFC					610 (25.9)	92 (1.9)	146 (3)	947 (16.7)	3504 (46.5)
< 60 ml/min per 1.73 m^2^					8 (< 1)	0	−10 (< 1)	105 (1.9)	711 (9.4)
< 30 ml/min per 1.73 m^2^					0	−1 (< 1)	−8 (< 1)	0	106 (1.4)
*N* (%) reclassified with BIS1									4322 (57.3)
< 60 ml/min per 1.73 m^2^									1447 (19.2)
< 30 ml/min per 1.73 m^2^									−39 (< 1)

BIS1, Berlin Initiative Study equation 1; CI, confidence interval; CKD-EPI, 2009 Chronic Kidney Disease Epidemiology Collaboration equation; CKiDU25, Chronic Kidney Disease in Children Study under 25; EKFC, European Kidney Function Consortium; IQR, interquartile range; PCr, plasma creatinine (μmol/L); ULN, upper limit of normal.

Results are expressed in median (IQR) or number (%).

Biases are expressed as median [95% CI].

Negative values indicate that the tested formula has fewer patients in the group than the reference formula.

#### Study Population

A total of 30,104 patients aged ≥ 2 years underwent PCr determination between November 1, 2023, and January 31, 2024. The population was divided into pediatric subgroups (children aged < 12 years, 12–15 years, and 15–18 years) and adult subgroups (young adults [aged 18–25 years], middle-aged adults [aged 25–40, 40–55, and 55–70 years], and older adults aged >70 years). The median age was 49.9 (43.8) years, with 49.8% male patients, and the median PCr was 66.0 (30.0) μmol/l. Among the entire population, 13.5% of patients (4060) had PCr values above the laboratory's upper normal limit for age and sex. As anticipated, median PCr values increased across the pediatric, adult, and elderly groups.

#### eGFR Results Using Different Formulas

Among the 25,364 adult patients, median eGFR-CKD-EPI was 93.7 (37.8) ml/min per 1.73 m^2^ and 15.2% (3854) of adults had an eGFR < 60 ml/min per 1.73 m^2^, and 3.5% (890) had an eGFR < 30 ml/min per 1.73 m^2^. eGFR-CKD-EPI was significantly higher than eGFR-EKFC (*P* < 0.0001) in the overall adult population and across all age subgroups. Median eGFR-EKFC remained relatively stable until the age of 40, after which it progressively decreased. The median bias between CKD-EPI and EKFC is lowest in the 40 to 55 years age group, at −2.1 (−2.2 to −2.1] ml/min per 1.73 m^2^; and highest in the 18 to 25 years age group at −16.3 (−16.5 to −16.0) ml/min per 1.73 m^2^.

Among the 4740 pediatric patients, the median eGFR-EKFC was 110.5 (15.4) ml/min per 1.73 m^2^ with 13.3% (632) having an eGFR < 90 ml/min per 1.73 m^2^ and 2% (95) < 60 ml/min per 1.73 m^2^. Because of the absence of height information in the laboratory database, eGFR was only calculated using EKFC in patients aged < 18 years.

For patients aged > 70 years (25.1% [7.543] patients), median eGFR-CKD-EPI was 72.2 [31.6] ml/min per 1.73 m^2^ and 33.3% (2512) of patients had an eGFR < 60 ml/min per 1.73 m^2^, and 6.5% (493) had an eGFR < 30 ml/min per 1.73 m^2^. eGFR-BIS1 was significantly lower than both eGFR-CKD-EPI and EKFC (*P* < 0.0001), with a median bias of −9.3 (−9.5 to −9.1] ml/min per 1.73 m^2^ compared with CKD-EPI and −1.9 (−2.1 to −1.8) ml/min per 1.73 m^2^ compared with EKFC.

**Figure 1:**
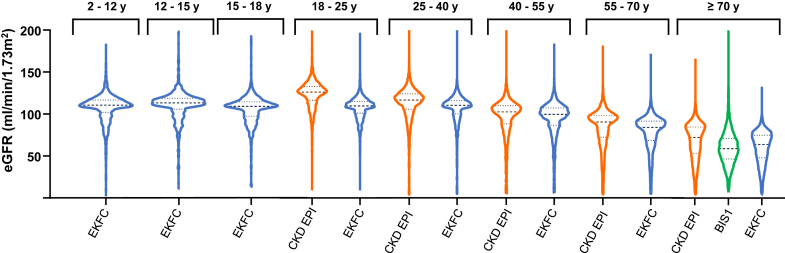
Distribution of eGFR in the whole population with EKFC (age-dependent and EKFC-height), CKD-EPI, and BIS1 equations according to age (violin plots). BIS1, Berlin Initiative Study 1; CKD-EPI, Chronic Kidney Disease Epidemiology Collaboration; eGFR, estimated glomerular filtration rate; EKFC, European Kidney Function Consortium; EKFC-height, EKFC equation with the inclusion of height.

In Figure 1, we show the distribution of eGFR using EKFC (whole population), CKD-EPI (in adults), and BIS1 (patients aged >70 years). In the age group >18 years, the CKD-EPI equation showed the highest values, with significant overestimation compared with other equations in both young adults and patients aged > 70 years.

#### CKD Stage Reclassification

Using CKD-EPI as the reference, we evaluated the proportion of adult patients reclassified to more or less severe CKD stages when applying the EKFC or BIS1 equations, both in the entire population and across different age groups. When EKFC was used, 25.1% of adult patients were reclassified, with 0.4% moving to a less severe stage and 24.7% to a more severe stage. Among these, 21% were reclassified from stage I to stage II, 3.3% from stage II to stage III, and 0.4% from stage III to stage IV.

In patients aged > 70 years, the number of reclassifications to more severe CKD stages was notably higher. Using EKFC, 9.4% moved from stage II to stage III, and 1.4% from stage III to stage IV. With BIS1, 19.2% moved from stage II to stage III, and 0.2% from stage III to stage IV. Notably, with EKFC compared with BIS1, no patients aged > 70 years were reclassified from stage II to stage III, whereas 2.1% were reclassified from stage III to stage IV.

In Supplementary Table S2, we display the frequency of KDIGO categories according to each eGFR equation. Regarding the frequency of KDIGO categories in the whole cohort, the concordance analysis between CKD-EPI and EKFC showed moderate agreement (κ = 0.76, 95% CI: 0.75–0.78). However, when stratified by age group, the agreement yielded Kappa coefficients ranging from a minimum of 0.58 (for adults aged ≥ 70 years) to a maximum of 0.88 (for adults aged 40–55 years), indicating almost substantial agreement among middle-aged adults, in contrast to lower agreement in young adults (κ = 0.65) and in those aged ≥ 70 years (κ = 0.58). In the group of older patients, the Kappa coefficient was similar between CKD-EPI and EKFC and between CKD-EPI and BIS1 (κ = 0.58).

### mGFR Substudy

#### Characteristics of the mGFR Substudy Population

In Table 2, we summarize the clinical characteristics, including age distribution and biological parameters, of patients with mGFR along with their classification according to KDIGO CKD stages.

**Table 2:** Description of the whole population and each age group characteristics of patient with mGFR (mGFR substudy)

	All	≥ 2 to < 12 yrs	≥ 12 to < 15 yrs	≥ 15 to < 18 yrs	≥ 18 to < 25 yrs	≥ 25 to < 40 yrs	≥ 40 to <55 yrs	≥ 55 to < 70 yrs	≥ 70 yrs
*n* (%)	4144	469	199	301	233	545	819	1111	467
Males (%)	2210 (53.3)	238 (50.7)	109 (54.8)	178 (59.1)	133 (57.1)	281 (51.6)	414 (50.5)	587 (52.8)	270 (57.8)
mGFR (ml/min per 1.73 m^2^)	71.5 (39.6)	102.1 (38.4)	93.3 (39.1)	88.7 (32.3)	79.4 (34.0)	76.8 (31.7)	73.0 (32.3)	59.2 (33.1)	44.2 (22.5)
Age (yrs)	47.1 (42.3)	7.5 (4.8)	13.7 (1.6)	16.6 (1.5)	20.7 (3.6)	32.8 (7.0)	48.6 (7.3)	62.0 (7.1)	74.5 (5.5)
Weight (kg)	65.0 (28.7)	23.3 (13.2)	44.8 (17.1)	57.0 (18.2)	60.0 (21.2)	67.1 (23.0)	72.0 (24.3)	71.0 (24.1)	73.0 (22.0)
Height (cm)	165.0 (16.0)	122.0 (28.0)	154.0 (14.4)	166.0 (12.5)	168.5 (13.3)	169.0 (14.0)	168.0 (13.0)	167.0 (14.0)	167.0 (12.0)
BSA (m^2^)	1.7 (0.4)	0.9 (0.4)	1.4 (0.3)	1.6 (0.3)	1.7 (0.3)	1.8 (0.4)	1.8 (0.3)	1.8 (0.3)	1.8 (0.3)
BMI (kg/m^2^)	23.3 (8.1)	15.9 (2.5)	18.7 (3.5)	20.7 (5.3)	21.4 (5.5)	23.4 (6.4)	25.1 (7.1)	25.4 (7.5)	25.8 (6.6)
PCr (μmol/l)	82.0 (51.5)	38.0 (21.0)	55.0 (31.0)	72.0 (33.3)	83.0 (34.5)	85.0 (39.5)	84.0 (45.0)	93.0 (55.0)	117.0 (56.8)
KDIGO classification, *n* (%)									
I	1042 (25.1)	310 (66.1)	111 (55.8)	142 (47.2)	68 (29.2)	145 (26.6)	174 (21.2)	88 (7.9)	4 (0.9)
II	1619 (39.1)	117 (24.9)	72 (36.2)	142 (41.2)	105 (45.1)	264 (484)	395 (48.2)	454 (40.9)	88 (18.8)
IIIa	688 (16.6)	31 (6.6)	10 (5.0)	24 (8.0)	34 (14.6)	70 (12.8)	127 (15.5)	259 (23.3)	133 (28.5)
IIIb	534 (12.9)	6 (1.3)	4 (2.0)	9 (3.0)	20 (8.6)	49 (59.0)	79 (9.6)	211 (19.0)	156 (33.4)
IV	250 (6.0)	5 (1.1)	2 (1.0)	2 (0.7)	6 (2.6)	16 (2.9)	41 (5.0)	96 (8.6)	82 (17.6)
V	11 (0.3)	0 (0)	0 (0)	0 (0)	0 (0)	1 (0.2)	3 (0.4)	3 (0.3)	4 (0.9)

BMI, body mass index; BSA, body surface area; IQR, interquartile range; KDIGO, Kidney Disease Improving Global Outcomes; mGFR, measured glomerular filtration rate; PCr: plasma creatinine.

Results are expressed in median (IQR) or *n* (%).

GFR was measured in 4144 patients. The median age was 47.1 (42.3) years with 53.3% male patients, median body mass index of 23.3 (8.1) kg/ m^2^, median PCr of 82.0 (51.5) μmol/l and a median mGFR of 71.5 (39.6) ml/min per 1.73 m^2^. The population was divided into pediatric subgroups (children: aged < 12 years, 12–15 years, and 15–18 years) and adult subgroups (young adults [aged 18–25 years], middle-aged adults [aged 25–40, 40–55, and 55–70 years], and older adults aged >70 years). Among the entire cohort, 23.4 % of measurements (969) were performed in patients aged < 18 years, 5.6% (233) in young adults (aged 18–25 years), and 11.2% (467) in patients aged >70 years. As expected in patients with mGFR, median PCr increased with age, whereas median mGFR decreased, and the prevalence of CKD increased (mGFR < 60 ml/min per 1.73 m^2^ in 9.6% (93) of patients aged < 18 years, 25.8% (59) in those aged 18–25 years, 25.0% (136) in those aged 25–40 years, 30.5% (569) in those aged 40 to 55 years, 51.2% (569) in those aged 55 to 70 years, and 79.7% (1628) in those aged > 70 years).

**Figure 2:**
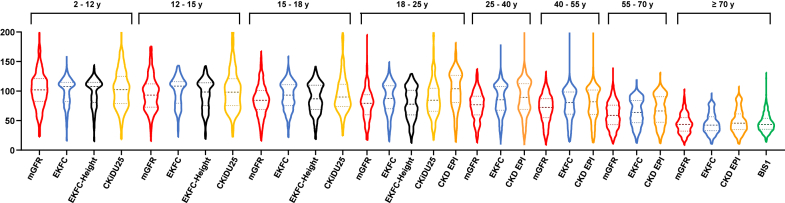
Distribution of GFR according to age groups and equations (mGFR, EKFC [age-dependent and EKFC-height], CKiDU25, CKD-EPI and BIS1) (violin plots). BIS1, Berlin Initiative Study 1; CKD-EPI, Chronic Kidney Disease Epidemiology Collaboration; CKiDU25, Chronic Kidney Disease in Children Under 25; EKFC, European Kidney Function Consortium; EKFC-height, EKFC equation with the inclusion of height; GFR, glomerular filtration rate; mGFR, measured GFR.

In Figure 2, we show the distribution of mGFR and eGFR in each age group (violin plots). The shapes of the distribution in the violin plot and the median differ dramatically between mGFR and CKD-EPI in young adults aged 18 to 25 years, with a marked overestimation of GFR by formulas as previously demonstrated.

**Figure 3:**
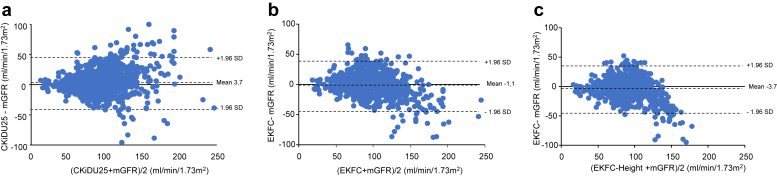
Bland Altman plots between mGFR and (a) CKiDU25, and (b) EKFC (age-dependent) and (c) EKFC-height in patients (aged < 18 years, *n* = 969). CKiDU25, Chronic Kidney Disease in Children Under 25; EKFC, European Kidney Function Consortium; EKFC-height, EKFC equation with the inclusion of height; mGFR, measured glomerular filtration rate.

**Figure 4:**
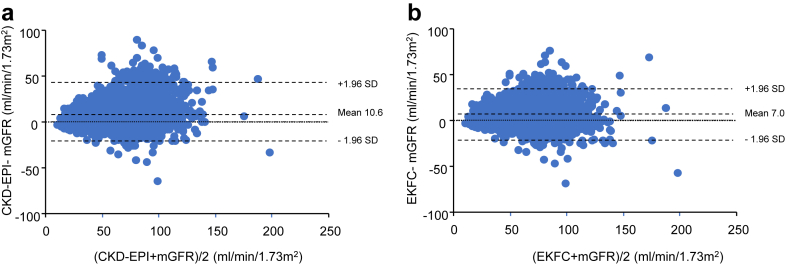
Bland Altman plots between mGFR and (a) CKD-EPI, and (b) EKFC in adults (aged ≥18 to < 70 years, *n* = 2708). CKD-EPI, Chronic Kidney Disease Epidemiology Collaboration; EKFC, European Kidney Function Consortium; mGFR, measured glomerular filtration rate.

**Figure 5:**
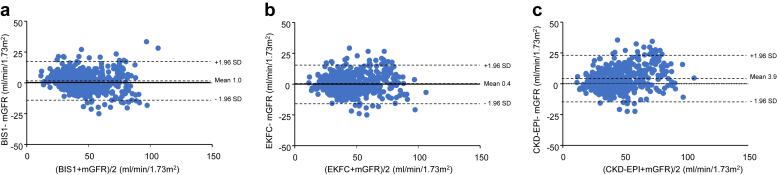
Bland Altman plots between mGFR and (a) BIS1, and (b) EKFC, and (c) CKD-EPI in older adults (aged > 70 years, *n* = 467). BIS1, Berlin Initiative Study 1; CKD-EPI, Chronic Kidney Disease Epidemiology Collaboration; EKFC, European Kidney Function Consortium; mGFR, measured glomerular filtration rate.

The Bland-Altman plots comparing eGFR values from different equations against mGFR for each patient group (children, adults, and elderly) are presented in Figures 3, 4, and 5. These plots highlight the wide dispersion of eGFR values, particularly at high or normal mGFR levels whatever the formula used.

#### Performance of the Various Equations Compared With mGFR (mGFR Substudy)

In Table 3, we summarize bias, imprecision, and accuracy with P30 and P10 between eGFR estimating equation and mGFR in the whole population and according to age groups. In children and adolescents, the median bias of the CKiDU25, EKFC, and EKFC-height equations was low, at 2.5 (1.2–3.9), −0.7 (−2.2 to 0.8) and −2.4 (−4.0 to −1.0) ml/min per 1.73 m^2^, respectively, with corresponding P30 accuracy of 85.7%,86.3%, and 89.1%. Both equations tended to underestimate eGFR in children aged < 12 years, whereas CKiDU25 and EKFC overestimate it in the 12 to 15 and 15 to 18-years subgroups. Although EKFC-height showed a lower bias in adolescents (1–18 years) compared with EKFC and CKiDU25, no significant differences were observed among the 3 equations in terms of accuracy, either in the overall pediatric population or within individual age groups.

**Table 3:** Performance of the various eGFR equations according to age groups (mGFR substudy)

	CKiDU25	EKFC-Height	EKFC	CKD-EPI	BIS-1	*P*
Pediatric patients (2–18 yrs) (*n* = 969)						
mGFR = 96.2 (38.8) ml/min per 1.73 m^2^						
eGFR, ml/min per 1.73 m^2^	97.0 (44.0)	99.3 (37.4)^a^^,^^b^	102.8 (34.5)^c^			
Bias, ml/min per 1.73 m^2^	2.5 (1.2–3.9)	−2.4 (−4.0 to 1.0)^a^^,^^b^	−0.7 (−2.2 to 0.8)^c^			< 0.001
Imprecision SD (P25–P75)	22.6 (-9.3 to 14.9)	20.0 (−13.4 to 8.2)	20.7 (−11.6 to 11.1)			< 0.001
Accuracy 10% (%)	38.7 (35.7–41.8)	42.7 (39.6–45.9)	41.4 (38.3–44.5)			0.186
Accuracy 30% (%)	85.7 (83.3–87.7)	89.1 (86.9–90.9)	86.3 (84.0–88.3)			0.060
Age group 1 (2 to < 12 yrs) (*n* = 469)						
mGFR= 102.1 (38.4) ml/min/1.73m^2^						
eGFR, ml/min per 1.73 m^2^	102.3 (46.0)	107.7 (33.8)^a^	107.5 (32.6)^c^			
Bias, ml/min per 1.73 m^2^	−0.4 (−2.6 to 2.1)	−4.7 (−6.3 to −2.7)^a^	−3.5 (−4.9 to −2.2)^c^			< 0.001
Imprecision SD (P25–P75)	22.8 (−12.3 to 12.8)	22.3 (−14.9 to 6.6)	23.0 (−15.8 to 8.3)			< 0.001
Accuracy 10% (%)	40.1 ( 35.7–44.6)	45.2 (40.8–49.7)	42.4 (38.0–46.9)			0.284
Accuracy 30% (%)	86.6 (83.2–89.4)	88.3 (85.0–90.9)	84.9 (81.3–87.8)			0.309
Age group (12 to < 15 yrs) (*n* = 199)						
mGFR = 93.3 (39.1) ml/min per 1.73 m^2^						
eGFR, ml/min per 1.73 m^2^	98.3 (45.0)	98.4 (38.5)^a^^,^^b^	108.4 (35.8)			
Bias, ml/min per 1.73 m^2^	5.1 (1.5–7.3)	−1.0 (−4.1 to 3.0)^a^^,^^b^	3.2 (−1.9 to 6.6)			< 0.001
Imprecision SD (P25–P75)	23.8 (−7.7 to 19.7)	17.9 (−13.0 to 9.9)	18.9 (−8.1 to 15.3)			< 0.001
Accuracy 10% (%)	35.7 (29.4–42.5)	40.7 (34.1–47.6)	39.7 (33.2–46.6)			0.553
Accuracy 30% (%)	83.4 (77.6–87.9)	89.4 (84.4–93.0)	83.4 (77.6–87.9)			0.144
Age group (15 to < 18 yrs) (*n* = 301)						
mGFR = 88.7 (32.3) ml/min per 1.73 m^2^						
eGFR, ml/min per 1.73 m^2^	89.8 (37.5)	86.8 (41.0)^a^^,^^b^	93.0 (35.0)			
Bias, ml/min per 1.73 m^2^	4.8 (2.3–7.1)	−0.6 (−1.6 to 1.2)^a^^,^^b^	2.9 (0.4–4.9)			< 0.001
Imprecision SD (P25–P75)	20.6 (−6.6 to 15.4)	16.9 (−12.0 to 10.3)	16.0 (−7.5 to 12.6)			< 0.001
Accuracy 10% (%)	38.5 (33.2–44.1)	40.2 (34.8–45.8)	40.9 (35.5–46.5)			0.835
Accuracy 30% (%)	85.7 (81.3–89.2)	90.0 (86.1–92.9)	90.4 (86.5–93.2)			0.132
Adult patients (*n* = 3175)						
mGFR = 64.6 (36.5) ml/min per 1.73 m^2^						
eGFR, ml/min per 1.73 m^2^			70.6 (43.3)	73.3 (47.0)		
Bias, ml/min per 1.73 m^2^			4.6 (4.2–5.1)	7.5 (6.9–8.1)		< 0.001
Imprecision SD (P25–P75)			13.5 (−2.9 to 13.6)	15.3 (−0.7 to 18.0)		< 0.001
Accuracy 10% (%)			38.1 (36.5–39.8)	33.9 (32.2–35.5)		< 0.001
Accuracy 30% (%)			82.2 (80.9–83.5)	75.2 (73.6–76.7)		< 0.001
Age group (18 to < 25 yrs) (*n* = 233)						
mGFR = 79.4 (34.0) ml/min per 1.73 m^2^						
eGFR, ml/min per 1.73 m^2^	84.6 (38.6)	77.9 (41.4)^b^	87.8 (38.7)^c^	103.8 (46.0)^d^^,^^e^^,^^f^		
Bias, ml/min per 1.73 m^2^	6.5 (4.4–9.1)	0.3 (−2.4 to 3.1)^b^	9.4 (5.1–12.1)^c^	21.9 (18.4–26.0)^d^^,^^e^^,^^f^		< 0.001
Imprecision SD (P25–P75)	20.3 (−2.1 to 21.0)	17.9 (−8.4 to 13.5)	17.3 (−3.2 to 21.4)	19.6 (8.2–36.3)		< 0.001
Accuracy 10% (%)	36.9 (31.0–43.3)	33.9 (28.1–40.2))	33.5 (27.7–39.8)	19.7 (15.1–25.3)		< 0.001
Accuracy 30% (%)	74.2 (68.3–79.4)	83.7 (78.4–87.9)	75.5 (69.6–80.6)	48.5 (42.2–54.9)		< 0.001
Age group (25 to < 40 yrs) (*n* = 545)						
mGFR = 76.8 (31.7) ml/min per 1.73 m^2^						
eGFR, ml/min per 1.73 m^2^			85.3 (40.1)	89.4 (43.0)		
Bias, ml/min per 1.73m^2^			9.7 (7.6–10.8)	12.2 (10.0–13.6)		< 0.001
Imprecision SD (P25–P75)			16.2 (0.2–19.8)	18.8 (2.5–24.1)		< 0.001
Accuracy 10% (%)			31.9 (28.2–36.0)	28.6 (25.0–32.6)		0.262
Accuracy 30% (%)			76.9 (73.2–80.2)	69.4 (65.4–73.1)		0.006
Age group (40 to < 55 yrs) (*n* = 819)						
mGFR = 73.0 (32.3) ml/min per 1.73 m^2^						
eGFR, ml/min per 1.73 m^2^			81.0 (37.4)	82.2 (41.3)		
Bias, ml/min per 1.73m^2^			6.3 (5.1–7.4)	7.3 (5.9–8.4)		< 0.001
Imprecision SD (P25–P75)			13.5 (−1.8–15.2)	14.9 (−1.7 to 17.5)		< 0.001
Accuracy 10% (%)			40.0 (36.7–43.4)	36.9 (33.6–40.2)		0.204
Accuracy 30% (%)			82.4 (79.7–84.9)	80.6 (77.7–83.1)		0.373
Age group (55 to < 70 yrs) (*n* = 1111)						
mGFR = 59.2 (33.1) ml/min per 1.73 m^2^						
eGFR, ml/min per 1.73 m^2^			64.2 (37.7)	66.4 (42.8)		
Bias, ml/min per 1.73 m^2^			3.8 (2.8–4.5)	6.7 (5.6–7.4)		< 0.001
Imprecision SD (P25–P75)			11.8 (−3.0–11.4)	12.9 (−1.1 to 15.6)		< 0.001
Accuracy 10% (%)			41.0 (38.1–43.9)	34.5 (31.7–37.3)		0.002
Accuracy 30% (%)			83.0 (80.7–85.1)	77.7 (75.1–80.0)		0.002
Age group (> 70 yrs) (*n* = 467)						
mGFR = 44.2 (22.5) ml/min per 1.73 m^2^						
eGFR, ml/min per 1.73 m^2^			42.9 (23.8)^e^	46.1 (26.5)	44.0 (18.8)^g^	
Bias, ml/min per 1.73 m^2^			−0.9 (−1.6 to 0.1)^e^	2.1 (0.9–3.1)	0.5 (−0.2 to 1.1)^g^	< 0.001
Imprecision SD (P25–P75)			8.4 (−5.1 to 5.8)	9.6 (−2.9–10.1)	8.2 (−4.3 to 6.3)	< 0.001
Accuracy 10% (%)			37.7 (33.4–42.2)	40.3 (35.9–44.8)	43.3 (38.8–47.8)	0.222
Accuracy 30% (%)			89.7 (86.6–92.2)	79.9 (76.0–83.3)	88.9 (85.7–91.4)	< 0.001

BIS1, Berlin Initiative Study equation 1; CI, confidence interval; CKD-EPI, 2009 Chronic Kidney Disease Epidemiology Collaboration equation; CKiDU25, Chronic Kidney Disease in Children Study under 25; eGFR : estimated GFR by creatinine by various formula; EKFC, European Kidney Function Consortium; IQR, interquartile range; mGFR; measured glomerular filtration rate by reference method.

Results of eGFR are presented as median (IQR).

Biases are expressed as median [95% CI].

Imprecision is expressed as SD of bias and interquartile range.

CKiDU25 as reference in young patients (< 25 yrs) and CKD EPI 2009 in adult patients (≥ 25 yrs).

*P* < 0.05:

a when comparing EKFC-height with CKIDU25,

b when comparing EKFC-height with EKFC,

c when comparing EKFC with CKIDU25,

d when comparing CKiDU25 with CKD-EPI,

e when comparing EKFC with CKD-EPI,

f when comparing EKFC-height with CKD-EPI, and

g when comparing BIS1 with CKD-EPI mGFR.

In adults, the performance of EKFC was better than CKD-EPI, with a median bias of 4.6 (4.2–5.1) versus 7.5 (6.9–8.1) ml/min per 1.73 m^2^ (*P* < 0.001) and a P30 accuracy of 82.2% versus 75.2% (*P* < 0.001). In young adults (aged 18–25 years), the CKD-EPI equation performed poorly, with a median bias of 21.9 (18.4–26.0) ml/min per 1.73 m^2^ and a P30 of 48.5%. The CKiDU25 and EKFC equations performed similarly in this age group, though suboptimally, with P30 values of 74.2% and 75.5 %, respectively. In contrast, EKFC-height performed best in young adults, showing a lower bias and a P30 accuracy of 83.7%. In adults aged 25 to 70 years, the EKFC equation shows lower bias and higher accuracy (P30) compared with the CKD-EPI equation (data not shown). This improvement is progressive, with decreasing bias and increasing performance from the 25 to 40 years age group to the 55–70 years group. However, both equations perform poorly in the 25 to 40 years age group, with respective biases of 9.7 and 12.2 ml/min per 1.73 m^2^ for EKFC and CKD-EPI, and P30 values of 76.9% and 69.4%. In the elderly population (aged > 70 years), the EKFC, CKD-EPI, and BIS1 equations showed low median biases of −0.9 (−1.6 to 0.1), 2.1 (0.9 to 3.1), and 0.5 (−0.2 to 1.1) ml/min per 1.73 m^2^, respectively, with corresponding P30 accuracy of 89.7%, 79.9%, and 88.9%.

In Supplementary Table S3 summarizes the performance of the various eGFR equations according to CKD stages. The performance of equations is studied according to GFR and groups of patients: children and adolescents (CKiDU25, EKFC, and EKFC-height), young adults (CKiDU25, EKFC, EKFC-height, and CKD-EPI), middle-aged adults (EKFC and CKD-EPI), and older patients (EKFC, CKD-EPI, and BIS). In children and adolescents, the CKiDU25, EKFC, and EKFC-height equations showed acceptable performance, with biases < 5 ml/min per 1.73 m^2^ and P30 values > 80%. The EKFC equation performed slightly better in individuals with normal kidney function. In young adults (aged 18–25 years), CKD-EPI overestimated mGFR in all patients. In this group of patients, the CKiDU25, EKFC, and CKD-EPI equations performed poorly, with notably low P30 values, except for the EKFC equation in stage I CKD. In contrast, EKFC-height performed best within CKD groups, showing a lower bias and a P30 accuracy of > 83% (except for stage IV and V group). The CKD-EPI equation, in particular, exhibited a very low P30 and a substantial positive bias regardless of the stage of CKD. In middle-aged patients, the EKFC equation yields lower bias and higher P30 compared with the CKD-EPI equation, although performance decreases with more advanced stages of CKD. In patients aged > 70 years, the CKD-EPI, EKFC, and BIS1 equations showed minimal bias, The EKFC and BIS1 equations achieved P30 values close to or the optimal 90% threshold. However, their performance decreased in advanced CKD (stages IV–V), with lower accuracy observed in this subgroup.

## Discussion

In this large-scale study, spanning the entire age spectrum and integrating 30,104 routine PCr measurements from the general population alongside 4144 mGFR values, we rigorously evaluated the following: (i) the impact of implementing the EKFC for eGFR in a general population and (ii) performance of 5 key eGFR equations (CKiDU25, EKFC, EKFC-height, CKD-EPI, and BIS1) in our local population. In the general population cohort (PCrAGE), implementation of the EKFC equation led to significant CKD stage reclassification using CKD-EPI as the reference, with 3.2% of individuals moving from stage II to stage III and 0.4% from stage III to stage IV. However, whereas CKD reclassification is rather minimal in the middle-aged population (aged 25–55 years), the proportion of reclassification is significant in both younger patients (aged < 25 years) and older patients (aged > 70 years). In children and adolescents, adopting EKFC instead of creatinine alone doubled the detection of individuals with probable GFR impairment. In the subset with mGFR (mGFR substudy), the accuracy of all eGFR equations remained suboptimal. Among adults, the EKFC equation consistently achieved the lowest bias and the highest P30 across age strata. In addition, in the adolescent and young adult groups, EKFC-height demonstrated the best performance. Nevertheless, performance for all equations declined with advancing CKD stages. Of note, CKD-EPI performed particularly poorly in young adults, whereas beyond the age of 70 years, all equations demonstrated comparable performance.

### General Population Considerations

Estimating GFR is essential for CKD diagnosis and management. Changes in GFR definitions can alter CKD prevalence and substantially reclassify patients into different CKD stages, impacting treatment strategies.^1^^,^^3^^,^^7^ CKD-EPI equations are applicable only to adults, meaning that any transition to the EKFC equation would primarily impact only patients aged > 18 years. In our adult study population, eGFR estimated using the CKD-EPI equation was significantly higher than that derived from the EKFC equation across all age groups. Transitioning to the EKFC equation would result in a sudden, unexplained decrease in eGFR values, potentially causing confusion for both patients and clinicians. However, reclassifying patients to a more severe stage could be valuable for screening purposes, particularly in specific subpopulations such as young adults or the elderly, by reducing the risk of false negatives. Nevertheless, this approach may result in a higher number of referrals to nephrologists, with both practical and economic implications. The performance of the EKFC and CKD-EPI equations was found to be comparable in previous studies involving middle-aged adults (25–70 years).^22^ In our study, within the subgroup of adults aged 25 to 70 years, the EKFC equation demonstrated lower bias and greater accuracy (P30) compared with the CKD-EPI equation, with both equations showing minimal bias in middle-aged individuals (40–55 years). However, their performance was substantially poorer in the 25 to 40 age group, with respective biases of 9.7 and 12.2 ml/min per 1.73 m^2^ for EKFC and CKD-EPI, and P30 values of 76.9% and 69.4%. The frequency of clinically significant CKD stage reclassification (e.g., stage II to III or III to IV) when switching from CKD EPI to EKFC is significant at the population level, occurring in 3.2% and 0.4% of the overall cohort, but remained low in just 0.6% and 0.0% of participants aged 25 to 70 years. Nevertheless, in our study, the overall performance of the various eGFR equations was poor, regardless of age group or CKD stage, likely reflecting a selection bias, because mGFR was obtained in chronic patients for whom estimation was expected to perform poorly, while reinforcing the notion that certain patients can currently only be accurately assessed through direct GFR measurement.

### Elderly Populations

The BIS1 and EKFC formulas were both applied in patients aged >r 70 years along with CKD-EPI. The BIS1 equation, first specifically designed for elderly patients by Schaeffner *et al.*,^16^ has shown variable performance compared with CKD-EPI. Although Koppe *et al.*^29^ reported better reliability of BIS1 in elderly patients, Vidal-Petiot *et al.*^18^ found similar bias and P30 values for BIS1 and CKD-EPI. Lopes *et al.*^17^ demonstrated that among creatinine-based equations, BIS1 performed best at mGFR < 60 ml/min per 1.73 m^2^, whereas CKD-EPI was superior at higher GFR levels. In our cohort of patients aged > 70 years of age with mGFR, the CKD-EPI, EKFC, and BIS1 equations exhibited minimal bias. Both the EKFC and BIS1 equations achieved P30 values approaching or exceeding the optimal 90% threshold, except in CKD stages IV–V. Overall, the CKD-EPI equation demonstrated lower performance, particularly in early and advanced stages of CKD, which contrasts with the findings reported by Lopes *et al.*^17^ This lower performance of the CKD-EPI equation in early stages of CKD likely explains why, in our general population cohort aged > 70 years, switching to the BIS1 equation would shift 19% of individuals from stage II to stage III, a change with potentially significant clinical implications. Although the overall performance of the EKFC and BIS1 equations in this older population was broadly similar, a key advantage of the EKFC equation over BIS1 lies in its ability to deliver a seamless and continuous estimation of GFR across the entire age spectrum, avoiding the discontinuities inherent to age-specific equations.

### Pediatric Populations

Despite current recommendations, many laboratories do not report eGFR in children, limiting early detection of CKD and undermining compliance with KDIGO guidelines.^3^^,^^7^ Earlier KDIGO guidelines^7^ endorsed the bedside creatinine-based CKD in Children 2009 equation for pediatric GFR estimation; and in 2020, Pierce *et al.* introduced the CKiDU25 equation for individuals aged < 25 years,^15^ improving accuracy but requiring height measurements and a formula change at the age of 25 years. Although height-dependent and age-dependent (height-independent) EKFC equations were developed concomitantly, with the height-dependent equation showing better performance in pediatric and young adult populations, the height-independent EKFC equation offers a seamless estimation of GFR from the age of 2 years through adulthood without requiring height measurements.^22^, ^23^, ^24^ In our laboratory database, EKFC identified subtle GFR impairments in pediatric populations, with 15.7% exhibiting an eGFR < 90 and 2% with < 60 ml/min per 1.73 m^2^, compared with only 7.5% detected by elevated PCr. This nearly 2-fold increase in detection underscores the clinical value of EKFC, particularly in routine laboratory settings where accurate height measurements are often unavailable. The performance of the CKiDU25 and EKFC equations in children, including specific age subgroups, has been shown in previous studies to be comparable in terms of precision and accuracy.^30^^,^^31^ Consistently, we observed low bias and high P30 for both equations in this age range. However, as previously noted by Nyman *et al.*,^30^ the CKiDU25 equation slightly outperforms EKFC in pediatric patients with CKD, which is consistent with the fact that it was specifically developed for this population. In addition, despite the slightly better performance of EKFC-height in this population, the need for height information prevents the use of this formula in laboratory routine.

### Young Adult Population

Young adults represented the most challenging subgroup in our study, because all 3 commonly used equations—CKiDU25, EKFC, and CKD-EPI—demonstrated poor performance, with notably low P30 values. The CKD-EPI equation, in particular, showed very low accuracy and a marked positive bias in individuals aged 18 to 25 years, indicating substantial overestimation of GFR, consistent with previous reports.^22^, ^23^, ^24^ In this context, adopting the EKFC equation in this age group, as well as CKiDU25 or EKFC-height when possible, may enable earlier identification of reduced kidney function, supporting timely implementation of follow-up strategies and nephroprotective interventions, while providing seamless continuity of eGFR assessment during the transition from pediatric to adult care.

### Limitations and Perspectives

One limitation of our study is its single-center design. Another limitation is that we evaluated eGFR estimating equations against mGFR in a population of patients with established or at-risk kidney disease, which does not strictly reflect the general population undergoing serum creatinine (PCr) testing in a routine laboratory setting. Due to the cohort size, some subgroups contained a limited number of individuals, particularly advanced CKD stages (IV or V) in younger patients and CKD stage 1 in older individuals, making it difficult to draw statistically robust conclusions for these subgroups. Finally, in the PCr study, we were unable to include the CKiDU25 or the EKFC-height equation because of the absence of height data; a constraint that reflects an inherent limitation of these equations.

There is a paucity of European studies addressing the use of eGFR equations, including EKFC, across the full age spectrum. Our large dataset (30,104 PCr and 4144 mGFR) provides valuable insights in this context and enables robust external validation. Strengths of our study include the use of a large laboratory database of PCr determinations (enzymatic method standardized to isotope dilution mass spectrometry), which could be generalized to a European population. In addition, the use of mGFR with a reference method in our own population, ranging from 2-year-old children to elderly patients, allows for the evaluation of the performance of creatinine-based eGFR equations compared with large studies.

## Conclusion

In this comprehensive, population-wide analysis combining > 30,000 PCr measurements and > 4000 directly measured GFR values, we provide a robust comparative assessment of widely used eGFR equations across the full age spectrum. The EKFC equation demonstrated consistent superiority in adults, yielding the lowest bias and highest accuracy across age strata, but with significant CKD stage reclassification in the general population especially in young adults and elderly. Importantly, given the similar performance of CKiDU25 and EKFC in children and adolescents, when height information is lacking, the use of EKFC in this population could significantly improve the detection of potential GFR impairment. Nevertheless, our findings highlight the persistent limitations of all creatinine-based equations, particularly in advanced CKD, and the notably poor performance of CKD-EPI in young adults, while reassuringly showing comparable performance of all equations in individuals aged > 70 years.

## Disclosure

All the authors declared no competing interests.

## Supplementary Material

Supplementary File (PDF) **Table S1.** Equations used to estimate glomerular filtration rate (ml/min per 1.73 m^2^). **Table S2.** Frequency of KDIGO CKD stages according to each eGFR equation (PCrAGE substudy). **Table S3.** Performance of the various eGFR equations across CKD stages, defined on the mean of measured GFR (mGFR) and estimated GFR (eGFR), with CKiDU25 applied to patients aged < 25 years and CKD-EPI to those aged > 25 years (mGFR substudy). Strobe checklist.

## Footnotes

Supplementary File (PDF)
**Table S1.** Equations used to estimate glomerular filtration rate (ml/min per 1.73 m^2^). **Table S2.** Frequency of KDIGO CKD stages according to each eGFR equation (PCrAGE substudy). **Table S3.** Performance of the various eGFR equations across CKD stages, defined on the mean of measured GFR (mGFR) and estimated GFR (eGFR), with CKiDU25 applied to patients aged < 25 years and CKD-EPI to those aged > 25 years (mGFR substudy). Strobe checklist.
