# Supplementary material for: Implementing Kidney Disease: Improving Global Outcomes 2024 Through Laboratory-Estimated GFR Across Ages
Source: Kidney Int Rep. 2026 Jan 3;11(3):103767. doi: 10.1016/j.ekir.2025.103767 (PMC12907000; doi:10.1016/j.ekir.2025.103767)
Supplement: Supplementary File (PDF) — Table S1. Equations used to estimate glomerular filtration rate (ml/min per 1.73 m2). Table S2. Frequency of KDIGO CKD stages according to each eGFR equation (PCrAGE substudy). Table S3. Performance of the various eGFR equations across CKD stages, defined on the mean of measured GFR (mGFR) and estimated GFR (eGFR), with CKiDU25 applied to patients aged < 25 years and CKD-EPI to those aged > 25 years (mGFR substudy). Strobe checklist. [file mmc1.pdf]

Supplementary materials.

Supplemental Table 1: Equations used to estimate glomerular filtration rate (mL/min/1.73m^2)

Name	GFR estimating equations (eGFR in mL/min/1.73 m^2)
**CKD-EPI***	$\text{Female; PCr} \leq 62, \text{eGFR} = 144 \text{ x} \left[\frac{\frac{\text{PCr}}{88.4}}{0.7}\right]^{-0.329} \text{ x } 0.9929^{\text{Age}} \text{ x [1.159 if black *]}$<br>$\text{Female; PCr} > 62, eGFR = 144 \text{ x}[\frac{\text{PCr/88.4}}{0.7}]^{-1.209} \text{x } 0.9929^{\text{Age}} \text{ x [1.159 if black *]}$<br>$\text{Male; PCr} \leq 80, \text{eGFR} = 141 \text{ x}[\frac{\text{PCr/88.4}}{0.9}]^{-0.411} \text{x } 0.9929^{\text{Age}} \text{x [1.159 if black *]}$<br>$\text{Male; PCr} > 80, eGFR = 141 \text{ x}[\frac{\text{PCr/88.4}}{0.9}]^{-1.209} \text{x } [0.9929]^{\text{Age}} \text{x [1.159 if black *]}$
**EKFC**	$2-40 \text{ years ; PCr/Q} < 1, \text{eGFR} = 107.3 \text{ x}[\frac{\text{PCr}}{Q}]^{-0.322}$<br>$2-40 \text{ years ; PCr/Q} \geq 1, \text{eGFR} = 107.3 \text{ x}[\frac{\text{PCr}}{Q}]^{-1.132}$<br>$> 40 \text{ years ; PCr/Q} < 1, \text{eGFR} = 107.3 \text{ x} \left[\frac{\text{PCr}}{Q}\right]^{-0.322} \text{ x } 0.990^{(\text{Age}-40)}$<br>$> 40 \text{ years ; PCr/Q} \geq 1, \text{eGFR} = 107.3 \text{ x} \left[\frac{\text{PCr}}{Q}\right]^{-1.132} \text{ x } 0.990^{(\text{Age}-40)}$<br>**With Q (µmol/L)**<br>$\text{Female; } 2-25 \text{ years ; ln(Q)} = 3.080 + 0.177 \text{ x } Age - 0.223\text{x ln}(Age) - 0.00596 \text{ x } Age^2 + 0.0000686 \text{ x } Age^3$<br>$\text{Male; } 2-25 \text{ years ; ln(Q)} = 3.2 + 0.259 \text{ x } Age - 0.543\text{x ln}(Age) - 0.00763 \text{ x } Age^2 + 0.0000790 \text{ x } Age^3$<br>$\text{Female; } \geq 25 \text{ years ; Q} = 62 \text{ µmol/L}$<br>$\text{Male; } \geq 25 \text{ years ; Q} = 80 \text{ µmol/L}$
**EKFC-Height**	$2-40 \text{ years ; PCr/Q} < 1, \text{eGFR} = 107.3 \text{ x}[\frac{\text{PCr}}{Q}]^{-0.322}$<br>$2-40 \text{ years ; PCr/Q} \geq 1, \text{eGFR} = 107.3 \text{ x}[\frac{\text{PCr}}{Q}]^{-1.132}$<br>**With Q (µmol/L)**<br>$\text{Q} = (3.94 - 13.4 \text{ x Ht} + 17.6 \text{ x Ht}^2 - 9.84 \text{ x Ht}^3 + 2.04 \text{ x Ht4}) \text{ x } 88.4 \text{ (Ht in m, Q in µmol/L)}$<br>$\text{Q} = 80 \text{ µmol/L when Ht} > 1.81 \text{ m}$
**BIS1**	$eGFR = 3736 \text{ x } (\frac{\text{PCr}}{88.4})^{-0.87} \text{ x } age^{-0.95} \times \text{[0.82 if female]}$
**CKiDU25**	$eGFR = \frac{\text{k x Ht}}{\text{Pcr/88.4}}$<br>$\text{Female; } < 12 \text{ years ; k} = 36.1 \text{ x } 1.008^{(\text{Age}-12)}$<br>$\text{Female; } 12-18 \text{ years ; k} = 36.1 \text{ x } 1.023^{(\text{Age}-12)}$<br>$\text{Female; } 18-25 \text{ years ; k} = 41.4$<br>$\text{Male; } < 12 \text{ years ; k} = 39.0 \text{ x } 1.008^{(\text{Age}-12)}$<br>$\text{Male; } 12-18 \text{ years ; k} = 39.0 \text{ x } 1.045^{(\text{Age}-12)}$<br>$\text{Male; } 18-25 \text{ years ; k} = 50.8$

Abbreviations: PCr: Plasma Creatinine (µmol/L); Age in years – Q values (µmol/L) correspond to the median plasma creatinine for the general population.
CKD-EPI: 2009 Chronic Kidney Disease–Epidemiology Collaboration; EKFC: European Kidney Function Consortium, BIS1: Berlin Initiative Study equation 1, CKiDU25: Chronic Kidney Disease in Children Study under 25.
* according to French recommendation the correction coefficient in Black people should not be used in European population(PMID: 36069913, PMID: 36002032). To convert creatinine values to mg/dL, multiply by 1/88.4.

Supplemental Table 2. Frequency of KDIGO CKD stages according to each eGFR equation (*PCrAGE Sub-Study)*.

**Adults patients**<br>ml/min/1.73m^2	**Hyperfiltration**<br>>135<br>N (%)	**G1:**<br>≥90<br>N (%)	**G2a:**<br>75 to <90<br>N (%)	**G2b:**<br>60 to <75<br>N (%)	**G3a:**<br>45 to <60<br>N (%)	**G3b:**<br>30 to <45<br>N (%)	**G4:**<br>15 to <30<br>N (%)	**G5:**<br><15<br>N (%)	**κ**	**95% CI**
**All patients (N = 25364)**										
**CKD EPI**	836 (3.3)	13212 (52.1)	4725 (18.6)	2737 (10.8)	1785 (7)	1179 (4.7)	586 (2.3)	304 (1.2)	0.76	0.75 to 0.78
**EKFC**	251 (1)	15588 (61.5)	5535 (21.8)	2450 (9.7)	716 (2.8)	407 (1.6)	244 (0.1)	173 (0.7)		
**≥18- <25 years (N=2359)**										
**CKD-EPI**	463 (19.6)	1813 (76.9)	44 (1.9)	19 (0.8)	10 (0.42)	4 (0.17)	3 (0.1)	3 (0.1)	0.65	0.54 to 0.75
**EKFC**	38 (1.6)	2079 (88.1)	185 (7.8)	29 (1.2)	17 (0.72)	5 (0.2)	3 (0.1)	3 (0.1)		
**≥25- <40 years (N=4891)**										
**CKD-EPI**	299 (6.1)	4066 (83.1)	290 (5.9)	101 (2.1)	40 (0.8)	40 (0.8)	21 (0.4)	34 (0.7)	0.83	0.78 to 0.87
**EKFC**	65 (1.3)	4196 (85.8)	374 (7.6)	121 (2.5)	44 (0.9)	37 (0.8)	21 (0.4)	33 (0.7)		
**≥40- <55 years (N=4904)**										
**CKD-EPI**	55 (1.1)	3522 (71.8)	673 (13.7)	299 (8.1)	151 (3.1)	81 (1.7)	60 (1.2)	63 (1.3)	0.88	0.83 to 0.93
**EKFC**	30 (0.6)	3453 (70.4)	752 (13.3)	324 (6.6)	155 (3.2)	75 (1.5)	62 (1.3)	53 (1.1)		
**≥55- <70 years (N=5667)**										
**CKD-EPI**	15 (0.3)	2905 (51.3)	1164 (20.5)	751 (13.3)	376 (6.6)	243 (4.3)	131 (2.3)	82 (1.4)	0.73	0.67 to 0.80
**EKFC**	5 (0.1)	1734 (30.6)	2047 (36.1)	944 (16.7)	454 (8)	270 (4.8)	139 (2.5)	74 (1.3)		
**≥70 years (N=7543)**										
**CKD-EPI***	4 (0.1)	906 (12)	2554 (33.9)	1567 (20.8)	1208 (16)	811 (10.8)	371 (4.9)	122 (1.6)	0.58	0.57 to 0.62
**EKFC***	0 (0)	131 (1.7)	1749 (23.2)	2440 (32.3)	1604 (21.3)	1020 (13.5)	472 (6.3)	127 (1.6)		
**BIS1**	26 (0.3)	443 (5.9)	1000 (13.3)	2115 (28)	225329.9)	1252 (16.6)	389 (5.2)	65 (0.9)	0.58	0.58 to 0.61

CKD-EPI: 2009 Chronic Kidney Disease–Epidemiology Collaboration; EKFC: European Kidney Function Consortium, BIS1: Berlin Initiative Study equation 1, CKiDU25 Chronic Kidney Disease in Children Study under 25.

Supplemental Table 3: performance of the various eGFR equations across CKD stages, defined on the mean of measured GFR (mGFR) and estimated GFR (eGFR), with

	Children and adolescents (< 18 years)			
	CKiDU25	EKFC-Height	EKFC	p
**All measurements**	**N = 969 (54.2% Males) Age : 12.4 (7.9) years (mGFR+eGFR)/2 : 97.1 (38.9) ml/min/1.73m^2**			
**Stage I (mGFR+eGFR)/2 > 90**	**N= 568 (52.8% Males Age : 11.6 (8.2) years (mGFR+eGFR)/2 : 112.5 (26.5) ml/min/1.73m^2**			
eGFR ml/min/1.73m^2	114.3 (34.0)	111.3 (12.4)$,§	112.5 (12.0)¤	p<0.0001
Bias ml/min/1.73m^2	5.2 [2.6;6.9]	-3.6 [-5.2;-1.7]$,§	-2.7 [-4.0;-1.2]¤	p<0.0001
Imprecision SD [P25;P75]	26.7 [-10.7; 20.4]	22.5 [-15.6; 8.5]	22.7 [-15.5; 9.9]	
Accuracy 10 % (%)	38.2 [34.3;42.3]	46.7 [42.6;50.8]	46.1 [42.1;50.2]	p=0.0047
Accuracy 30 % (%)	84.0 [80.7;86.8]	91.4 [88.8 ; 93.4]	89.1 [86.3;91.4]	p=0.0009
**Stage II 60 > (mGFR+eGFR)/2 ≤ 90**	**N= 325 (56.3% Males) Age :13.2 (7.3) years (mGFR+eGFR)/2 :78.3 (13.6) ml/min/1.73m^2**			
eGFR ml/min/1.73m^2	76.2 (14.1)	75.5 (17.5)$,§	79.9 (19.4)¤	p<0.0001
Bias ml/min/1.73m^2	-0.9 [-2.7;2.1]	-0.6 [-2.7;1.5]$,§	2.8 [0.6 ;5.2]¤	p<0.0001
Imprecision SD [P25;P75]	15.4 [-9.2; 9.6]	16.2 [-11.4; 9.2]	17.3 [-7.9; 14.7]	
Accuracy 10 % (%)	41.2 [36.0;46.7]	38.8 [33.6;44.2]	36.3 [31.3;41.7]	p=0.1687
Accuracy 30 % (%)	88.9 [85.0;91.9]	86.5 [82.3 ; 89.8]	84.3 [80.0;87.9]	p=0.0088
**Stage III 30 ≥ (mGFR+eGFR)/2 < 60**	**N= 65 (52.3% Males) Age: 13.0(7.1) years (mGFR+eGFR)/2 : 53.1(9.8) ml/min/1.73m^2**			
eGFR ml/min/1.73m^2	51.6(12.2)	49.0 (13.5)$,§	54.1 (16.0)	p<0.0001
Bias ml/min/1.73m^2	1.0 [-2.4 ;5.5]	-1.8 [-6.3;0.9]$,§	2.5 [-2.8 ; 6.4]	p<0.0001
Imprecision SD [P25;P75]	10.1 [-6.6; 7.8]	11.1 [-10.0; 5.9]	11.5 [-5.7; 10.8]	
Accuracy 10 % (%)	30.8 [20.9;42.8]	29.2 [19.6;41.2]	29.2 [19.6;41.2]	p=0.7568
Accuracy 30 % (%)	86.1 [75.7; 92.5]	86.2 [75.7 ; 92.5]	80.0 [68.7;87.9]	p=0.1293
**Stage IV-V (mGFR+eGFR)/2 < 30**	**N= 11 (72.7% Males) Age: 12.0 (8.1) years (mGFR+eGFR)/2 : 25.5 (5.7) ml/min/1.73m^2**			
eGFR ml/min/1.73m^2	23.7(6.7)	19.6 (5.5)$	21.4(9.0)	p=0.0084
Bias ml/min/1.73m^2	0.7 [-7.2 ; 6.9]	-3.1 [-12.8;1.1]$	-3.7 [-9.5 ; 6.9]	p=0.0084
Imprecision SD [P25;P75]	6.4 [-5.6; 5.8]	6.2 [9.0;0.3]	7.8 [-8.8; 6.5]	
Accuracy 10 % (%)	36.4 [15.2; 64.6]	36.4 [15.2;64.6]	18.2 [3.2;47.7]	p=0.631
Accuracy 30 % (%)	72.7 [43.4 ;90.3]	63.6 [35.4 ; 84.8]	36.4[15.2;64.6]	p=0.3426

	Adults (> 18 years)		
	EKFC	CKD-EPI	p
**All measurements**	**N= 3175 (53.1% Males) Age :54.9 (24.6) years (mGFR+eGFR)/2: 69.2 (40.1) ml/min/1.73m^2**		
**Stage I (mGFR+eGFR)/2 > 90**	**N= 739(52.0% Males) Age: 42.8(22.1) years (mGFR+eGFR)/2 :99.5 (12.6) ml/min/1.73m^2**		
eGFR ml/min/1.73m^2	103.9 (14.8)	108.2 (17.2)	p<0.0001
Bias ml/min/1.73m^2	9.5 [7.7;10.7]	14.7 [13.4;16.5]	p<0.0001
Imprecision SD [P25;P75]	16.2 [-0.4; 18.9]	18.2 [5.4; 25.8]	
Accuracy 10 % (%)	42.5 [39.0;46.1]	33.7 [30.4;37.2]	p=0.0006
Accuracy 30 % (%)	86.3 [83.7;88.6]	75.6 [72.4;78.6]	p<0.0001
**Stage II 60 > (mGFR+eGFR)/2 ≤ 90**	**N= 1227 (51.0% Males Age: 53.7(22.5) years (mGFR+eGFR)/2 :75.5 0(14.8) ml/min/1.73m^2**		
eGFR ml/min/1.73m^2	77.5 (17.2)	81.2 (19.9)	p<0.0001
Bias ml/min/1.73m^2	6.3 [5.3;7.3]	10.6 [9.5;11.5]	p<0.0001
Imprecision SD [P25;P75]	14.4 [-2.4; 16.3]	15.6 [1.2; 20.9]	
Accuracy 10 % (%)	38.9 [36.2;41.6]	32.1 [29.6;34.8]	p=0.005
Accuracy 30 % (%)	80.4 [78.1;82.6]	72.5 [69.9;74.9]	p<0.0001
**Stage III 30 ≥ (mGFR+eGFR)/2 < 60**	**N= 1003 (57.0% Males) Age: 63.0(19.0) years (mGFR+eGFR)/2 :46.2 (14.4) ml/min/1.73m^2**		
eGFR ml/min/1.73m^2	46.2(15.7)	47.7(16.7)	p<0.0001
Bias ml/min/1.73m^2	1.8 [1.0; 2.5]	3.0 [2.4; 3.6]	p<0.0001
Imprecision SD [P25;P75]	9.9 [-3.8; 8.5]	10.5 [-2.8; 9.8]	
Accuracy 10 % (%)	36.4 [33.5;39.4]	37.9 [34.9;40.9]	p=0.5177
Accuracy 30 % (%)	82.6 [80.1 ;84.8]	78.1 [75.4; 80.5]	p=0.0134
**Stage IV-V (mGFR+eGFR)/2 < 30**	**N= 206 (50.0% Males) Age :63.7 (20.6) years (mGFR+eGFR)/2 : 24.8(6.3) ml/min/1.73m^2**		
eGFR ml/min/1.73m^2	24.8(6.6)	24.4 (7.5)	p=0.0003
Bias ml/min/1.73m^2	0.3 [-1.0;1.6]	0.1 [-0.9;1.3]	p=0.0003
Imprecision SD [P25;P75]	6.8 [-3.7; 5.4]	7.0 [-4.0; 5.3]	
Accuracy 10 % (%)	26.7 [21.1;33.1]	25.2 [19.8;31.6]	p=0.8223
Accuracy 30 % (%)	76.7 [70.5;82.0]	75.7 [69.4;81.1]	p=0.9079

	Young adults (18- 25 years)				
	CKiDU25	EKFC-Height	EKFC	CKD-EPI	p
**All measurements**	**N= 233 (57.1% Males) Age :20.7 (3.6) years (mGFR+eGFR)/2 : 81.6 (33.2) ml/min/1.73m^2**				
**Stage I (mGFR+eGFR)/2 > 90**	**N= 95 (56.8% Males) Age: 20.5(4.1) years (mGFR+eGFR)/2: 101.0(18.7) ml/min/1.73m^2**				
eGFR ml/min/1.73m^2	107.8 (27.0)	107.3 (16.7)§	110.2(12.8)	128.5 (14.2)#,*,$	p<0.0001
Bias ml/min/1.73m^2	12.9 [5.6;22.3]	5.2 [0.2;12.7]§	13.9 [7.6;17.1]	32.0 [25.4;35.8]#,*,$	p<0.0001
Imprecision SD [P25;P75]	25.6 [-2.1; 34.5]	21.1 [-9.5;18.2]	19.1 [-3.4; 24.3]	19.3 [14.3; 43.3]	
Accuracy 10 % (%)	34.7[25.9;44.7]	34.7 [25.9;44.7]	32.6[24.0;42.6]	15.8 [9.8;24.3]	p=0.0344
Accuracy 30 % (%)	66.3[56.3;75.0]	84.2 [75.6 ; 90.2]	77.9 [68.6;85.0]	46.3[36.6;56.3]	p=0.0001
**Stage II 60 > (mGFR+eGFR)/2 ≤ 90**	**N= 93 (59.1% Males) Age: 20.9 (3.3) years (mGFR+eGFR)/2 :75.5 (13.5))**				
eGFR ml/min/1.73m^2	76.1 (15.5)	70.7 (18.1)§	80.1 (16.3)	91.0 (22.2)#,*,$	p<0.0001
Bias ml/min/1.73m^2	5.5 [1.1; 9.1]	-2.3 [-5.2;2.1]§	6.8 [3.0;13.3]	19.1 [14.8; 26.0]#,*,$	p<0.0001
Imprecision SD [P25;P75]	15.7 [-5.8; 13.7]	17.2 [-11.2; 9.9]	16.4 [-3.6; 19.9]	18.9 [7.2; 33.5]	
Accuracy 10 % (%)	36.6 [27.5;46.7]	34.4 [25.5;44.5]	38.7 [29.4;48.9]	22.6 [15.3;32.1]	p=0.0707
Accuracy 30 % (%)	81.7 [72.7;88.3]	83.9 [75.1 ; 90.0]	75.3 [65.6;82.9]	49.5 [39.5;59.4]	p<0.0001
**Stage III 30 ≥ (mGFR+eGFR)/2 < 60**	**N= 40 (52.5% Males) Age: 20.5 (2.8) years (mGFR+eGFR)/2 :48.9 (15.9))**				
eGFR ml/min/1.73m^2	53.3 (17.2)	45.6 (17.5)§	53.7(21.3)	60.6 (26.4)#,*,$	p<0.0001
Bias ml/min/1.73m^2	4.1 [0.9 ;8.1]	-2.2 [-6.1;3.6]§	5.1 [2.0; 9.8]	12.4 [4.4;18.5]#,*,$	p<0.0001
Imprecision SD [P25;P75]	12.1 [-0.2; 11.3]	10.5 [-7.1; 5.3]	15.5 [-1.5; 16.7]	18.8 [3.0; 24.4]	
Accuracy 10 % (%)	45.0 [30.7; 60.2]	35.0 [22.1;50.5]	25.0 [14.2;40.2]	25.0 [14.2;40.2]	p=0.0674
Accuracy 30 % (%)	80.0 [65.2; 89.5]	85.0 [70.9 ; 92.9]	72.5 [57.2;83.9]	50.0 [35.2;64.8]	p<0.0001
**Stage IV-V (mGFR+eGFR)/2 < 30**	**N= 5 (60.0% Males) Age: 23.2 (1.9) years (mGFR+eGFR)/2 :25.2 (6.9))**				
eGFR ml/min/1.73m^2	27.4 (11.2)	23.4 (9.6)	24.8 (13.9)	25.5(15.0)$	p=0.023
Bias ml/min/1.73m^2	5.5[-1.8;18.9]	2.5 [-6.3;12.5]	2.5 [-3.8;22.8]	2.6 [-4.6;25.5]$	p=0.023
Imprecision SD [P25;P75]	7.7 [1.4; 14.5]	6.7[-2.0; 7.9]	10.2 [-0.9; 15.8]	11.4 [-1.1; 17.2]	
Accuracy 10 % (%)	20.0 [1.0;62.4]	0.0 [0.0;43.4]	20.0 [1.0;62.4]	0.0 [0;43.4]	p=0.5708
Accuracy 30 % (%)	40.0 [7.1;76.9]	60.0 [23.1 ; 92.9]	60.0 [23.1;92.9]	60.0 [23.1; 92.9]	p=0.8435

	Middle-aged Adults (25-70 years)		
	EKFC	CKD-EPI	p
**All measurements**	**N= 2475 (51.8% Males) Age :53.1 (19.5) years (mGFR+eGFR)/2 : 73.4(36.9) ml/min/1.73m^2**		
**Stage I (mGFR+eGFR)/2 > 90**	**N= 639 (51.5% Males) Age: 45.4(18.8) years (mGFR+eGFR)/2: 99.3(12.1) ml/min/1.73m^2**		
eGFR ml/min/1.73m^2	102.8 (14.1)	107.0 (14.3)	p<0.0001
Bias ml/min/1.73m^2	9.3 [7.6; 10.5]	13.5 [12.0;14.8]	p<0.0001
Imprecision SD [P25;P75]	15.7 [0.1; 18.7]	17.3 [4.5; 22.6]	
Accuracy 10 % (%)	43.8 [40.0;47.7]	36.2 [32.5;39.9]	p=0.0061
Accuracy 30 % (%)	87.5 [84.7;89.8]	79.8 [76.5;82.7]	p=0.0003
**Stage II 60 > (mGFR+eGFR)/2 ≤ 90**	**N= 1029 (50.0% Males) Age: 53.5(18.5) years mGFR+eGFR)/2 : 75.9(14.9) ml/min/1.73m^2**		
eGFR ml/min/1.73m^2	78.0 (17.6)	80.6 (20.1)	p<0.0001
Bias ml/min/1.73m^2	6.7 [5.7;7.8]	9.8 [8.7; 11.0]	p<0.0001
Imprecision SD [P25;P75]	14.6 [-2.3; 16.3]	15.4 [0.0; 19.7]	
Accuracy 10 % (%)	38.1 [35.2;41.1]	32.6 [29.8;35.5]	p :0.0098
Accuracy 30 % (%)	79.7 [77.1;82.0]	74.2 [71.5;76.8]	p : 0.004
**Stage III 30 ≥ (mGFR+eGFR)/2 < 60**	**N= 676 (56.2% Males) Age: 58.6(14.3) years mGFR+eGFR)/2 : 47.2 (14.5) ml/min/1.73m^2**		
eGFR ml/min/1.73m^2	48.1 (15.1)	48.7 (16.5)	p<0.0001
Bias ml/min/1.73m^2	3.3 [2.2; 4.1]	3.5 [2.8; 4.2]	p<0.0001
Imprecision SD [P25;P75]	9.7 [-2.6; 9.9]	10.0 [-2.6; 10.3]	
Accuracy 10 % (%)	37.1 [33.6;40.8]	35.8 [32.3;39.5]	p :0.6513
Accuracy 30 % (%)	79.6 [76.4;82.5]	78.1 [74.8 ;81.1]	p : 0.5490
**Stage IV-V (mGFR+eGFR)/2 < 30**	**N= 131 (45.0% Males) Age: 57.9 (14.9) years mGFR+eGFR)/2 : 24.6 (5.7) ml/min/1.73m^2**		
eGFR ml/min/1.73m^2	25.2 (7.0)	24.1(7.4)	p<0.0001
Bias ml/min/1.73m^2	0.8 [-1.0; 2.5]	-0.2 [-1.7; 1.7]	p<0.0001
Imprecision SD [P25;P75]	7.1 [-3.6; 5.7]	7.1 [-4.8; 5.2]	
Accuracy 10 % (%)	26.0 [19.2;34.1]	25.2 [18.5 ;33.3]	p >0.9999
Accuracy 30 % (%)	75.6 [67.6;82.1]	75.6 [67.6;82.1]	p >0.9999

	Older adults (>70 years)			
	EKFC	CKD-EPI	BIS-1	p
**All measurements**	**N= 467 (57.8% Males) Age: 74.5(5.5) years (mGFR+eGFR)/2: 45.2 (24.2) ml/min/1.73m^2**			
**Stage I (mGFR+eGFR)/2 > 90**	**N= 5 (20.0 % Males) Age: 73.4(3.1) years mGFR+eGFR)/2:96.9 (8.9) ml/min/1.73m^2**			
eGFR ml/min/1.73m^2	82.4 (12.0)*	92.0 (15.2)	85.0 (44.5)	p=0.17
Bias ml/min/1.73m^2	-8.3 [-20.7;3.5]*	0.5 [-11.1;15.4]	-9.2 [-18.1;33.1]	p=0.17
Imprecision SD [P25;P75]	8.8 [-16.5; -1.6]	9.9 [-7.7; 10.0]	24.9 [-17.1; 30.5]	
Accuracy 10 % (%)	60.0 [23.1;92.9]	60.0 [23.1;92.9]	0.0 [0.0;43.4]	p=0.0821
Accuracy 30 % (%)	100.0 [56.6;100.0]	100.0[56.6;100.0]	80.0 [37.6;99.0]	p=0.3425
**Stage II 60 > (mGFR+eGFR)/2 ≤ 90**	**N= 105 (54.3 % Males) Age: 73.9(4.2) years mGFR+eGFR)/2 : 71.6 (12.8) ml/min/1.73m^2**			
eGFR ml/min/1.73m^2	69.7 (14.4)*	76.9 (17.3)	64.6 (12.8)£	p<0.0001
Bias ml/min/1.73m^2	4.5 [1.3; 6.6]*	11.1 [8.9;14.5]	-0.3 [-2.7; 2.7]£	p<0.0001
Imprecision SD [P25;P75]	9.5 [-3.4; 11.3]	30.4 [2.7; 18.9]	9.5 [-7.4; 6.7]	
Accuracy 10 % (%)	46.7 [37.4;56.2]	36.2 [27.6;45.7]	49.5 [40.1;58.9]	p=0.1226
Accuracy 30 % (%)	92.4 [85.7;96.1]	75.2 [66.2;82.5]	97.1 [91.9;99.2]	p<0.0001
**Stage III 30 ≥ (mGFR+eGFR)/2 < 60**	**N= 287 (59.6 % Males) Age: 74.6 (5.7) years mGFR+eGFR)/2 : 43.6 (12.3) ml/min/1.73m^2**			
eGFR ml/min/1.73m^2	41.4 (12.4)*	43.8 (13.6)	42.9 (10.1)£	p<0.0001
Bias ml/min/1.73m^2	-1.8 [-2.9;-0.9]*	0.5 [-0.1;2.1]	-0.1 [-1.1;0.8]£	p<0.0001
Imprecision SD [P25;P75]	8.1 [-5.9; 4.7]	8.7 [-3.2; 7.8]	7.5 [-4.9; 5.7]	
Accuracy 10 % (%)	36.2 [30.9;41.9]	44.6 [38.9;50.4]	43.9 [38.3;49.7]	p=0.0786
Accuracy 30 % (%)	90.9 [87.1;93.7]	81.9 [77.0;85.9]	90.2 [83.3;93.2]	p=0.007
**Stage IV-V (mGFR+eGFR)/2 < 30**	**N= 70 (58.6 % Males) Age: 75.6 (7.9) years mGFR+eGFR)/2 : 25.0(7.2) ml/min/1.73m^2**			
eGFR ml/min/1.73m^2	24.1 (6.0)*	24.6 (7.0)	28.4 (5.4)£	p<0.0001
Bias ml/min/1.73m^2	-0.8 [-2.8; 1.1]*	0.1 [-2.1;2.0]	3.7 [1.7;4.6]£	p<0.0001
Imprecision SD [P25;P75]	5.9 [-3.9; 4.0]	6.2 [-3.8; 5.2]	5.6 [0.4; 7.3]	
Accuracy 10 % (%)	28.6 [19.3;40.1]	27.1 [18.1;38.5]	34.3 [24.2;46.0]	p=0.6211
Accuracy 30 % (%)	80.0 [69.2;87.7]	77.1 [66.1;85.4]	71.4 [56.0;80.7]	p=0.4795

CKiDU25 applied to patients under 25 years and CKD-EPI to those over 25 years (*mGFR sub-Study)*.

Results of eGFR are presented as median (IQR). Bias are expressed as median [95%CI]. Imprecision is expressed as SD of bias and interquartile range. Accuracy 10 and 30% are expressed as percentage[95%CI]. CKD stage were stratified by the average of mGFR and eGFR, using age-appropriate equations (CKiD-U25 <25 years; EKFC ≥25 years).¤ p<0.05 when comparing EKFC with CKIDU25; # p<0.05 when comparing CKiDU25 with CKD-EPI;£ p<0.05 when comparing BIS1 with CKD-EPI, * when comparing EKFC with CKD-EPI, $ when comparing EKFC-Height with CKIDU25, x when comparing EKFC-Height with CKD-EPI, § when comparing EKFC-Height with EKFC .mGFR: measured GFR by reference method;eGFR : estimated GFR by creatinine by various formulas.

STROBE Statement—checklist of items that should be included in reports of observational studies

	Item No	Recommendation
**Title and abstract**	1	(*a*) Indicate the study's design with a commonly used term in the title or the abstract
		(*b*) Provide in the abstract an informative and balanced summary of what was done and what was found
**Introduction**		
Background/rationale	2	Explain the scientific background and rationale for the investigation being reported
Objectives	3	State specific objectives, including any prespecified hypotheses
**Methods**		
Study design	4	Present key elements of study design early in the paper
Setting	5	Describe the setting, locations, and relevant dates, including periods of recruitment, exposure, follow-up, and data collection
Participants	6	(*a*) *Cohort study*—Give the eligibility criteria, and the sources and methods of selection of participants. Describe methods of follow-up<br>*Case-control study*—Give the eligibility criteria, and the sources and methods of case ascertainment and control selection. Give the rationale for the choice of cases and controls<br>*Cross-sectional study*—Give the eligibility criteria, and the sources and methods of selection of participants
		(*b*) *Cohort study*—For matched studies, give matching criteria and number of exposed and unexposed<br>*Case-control study*—For matched studies, give matching criteria and the number of controls per case
Variables	7	Clearly define all outcomes, exposures, predictors, potential confounders, and effect modifiers. Give diagnostic criteria, if applicable
Data sources/ measurement	8*	For each variable of interest, give sources of data and details of methods of assessment (measurement). Describe comparability of assessment methods if there is more than one group
Bias	9	Describe any efforts to address potential sources of bias
Study size	10	Explain how the study size was arrived at
Quantitative variables	11	Explain how quantitative variables were handled in the analyses. If applicable, describe which groupings were chosen and why
Statistical methods	12	(*a*) Describe all statistical methods, including those used to control for confounding
		(*b*) Describe any methods used to examine subgroups and interactions
		(*c*) Explain how missing data were addressed
		(*d*) *Cohort study*—If applicable, explain how loss to follow-up was addressed<br>*Case-control study*—If applicable, explain how matching of cases and controls was addressed<br>*Cross-sectional study*—If applicable, describe analytical methods taking account of sampling strategy
		(*e*) Describe any sensitivity analyses

Continued on next page

**Results**		
Participants	13*	(a) Report numbers of individuals at each stage of study—eg numbers potentially eligible, examined for eligibility, confirmed eligible, included in the study, completing follow-up, and analysed
		(b) Give reasons for non-participation at each stage
		(c) Consider use of a flow diagram
Descriptive data	14*	(a) Give characteristics of study participants (eg demographic, clinical, social) and information on exposures and potential confounders
		(b) Indicate number of participants with missing data for each variable of interest
		(c) *Cohort study*—Summarise follow-up time (eg, average and total amount)
Outcome data	15*	*Cohort study*—Report numbers of outcome events or summary measures over time
		Case-control study—Report numbers in each exposure category, or summary measures of exposure
		Cross-sectional study—Report numbers of outcome events or summary measures
Main results	16	(*a*) Give unadjusted estimates and, if applicable, confounder-adjusted estimates and their precision (eg, 95% confidence interval). Make clear which confounders were adjusted for and why they were included
		(*b*) Report category boundaries when continuous variables were categorized
		(*c*) If relevant, consider translating estimates of relative risk into absolute risk for a meaningful time period
Other analyses	17	Report other analyses done—eg analyses of subgroups and interactions, and sensitivity analyses
**Discussion**		
Key results	18	Summarise key results with reference to study objectives
Limitations	19	Discuss limitations of the study, taking into account sources of potential bias or imprecision. Discuss both direction and magnitude of any potential bias
Interpretation	20	Give a cautious overall interpretation of results considering objectives, limitations, multiplicity of analyses, results from similar studies, and other relevant evidence
Generalisability	21	Discuss the generalisability (external validity) of the study results
**Other information**		
Funding	22	Give the source of funding and the role of the funders for the present study and, if applicable, for the original study on which the present article is based

*Give information separately for cases and controls in case-control studies and, if applicable, for exposed and unexposed groups in cohort and cross-sectional studies.

Note: An Explanation and Elaboration article discusses each checklist item and gives methodological background and published examples of transparent reporting. The STROBE checklist is best used in conjunction with this article (freely available on the Web sites of PLoS Medicine at http://www.plosmedicine.org/, Annals of Internal Medicine at http://www.annals.org/, and Epidemiology at http://www.epidem.com/). Information on the STROBE Initiative is available at www.strobe-statement.org.
